# Effect of Modulated Masking on Cortical Auditory Evoked Potential in Normal Hearing Individuals: A Systematic Review and Meta-analysis

**DOI:** 10.1055/s-0044-1782629

**Published:** 2024-04-12

**Authors:** Mônyka Ferreira Borges Rocha, Karina Paes Advíncula, Jéssica Dayane da Silva, Pedro de Lemos Menezes

**Affiliations:** 1 Department of Speech Therapy, Universidade Federal de Alagoas, Maceió, AL, Brazil; 2 Department of Speech Therapy, Universidade Federal de Pernambuco, Recife, PE, Brazil; 3 Department of Human Communication Health, Universidade Federal de Pernambuco, Recife, PE, Brazil; 4 Department of Speech Therapy, Universidade Estadual de Ciências da Saúde de Alagoas, Maceió, AL, Brazil; 5 Department of Biotechnology, Universidade Federal de Alagoas, Maceió, AL, Brazil

**Keywords:** electrophysiology, auditory evoked potentials, noise, speech perception, perceptual masking, hearing

## Abstract

**Introduction** The study of electrophysiological auditory measures with different types of masking makes it possible to understand temporal processing skills and the processes involved in speech recognition in noise situations. The use of modulated masking in cortical measures of hearing enables the obtainment of analysis parameters of the masking release and its impact on neural auditory processing.

**Objective** To investigate the behavior of cortical auditory evoked potentials (CAEPs) with modulated masking in the normal hearing population.

**Data synthesis** A total of 2,159 articles were identified in the initial search; of these, 12 were selected for full reading. After excluding studies that did not meet the eligibility criteria, six articles were included in the present systematic review.

The results show that the type of masking has an influence on cortical auditory behavior, indicating a different effect on neural posture rergarding CAEP responses. Modulated noise as masking in the CAEP record generated statistically higher and earlier responses compared with non-modulated/steady noise, evidenced by the results obtained in the meta-analysis with subgroup analysis. These responses may indicate an influence of the type of noise in the neural auditory coding.

**Conclusion** Better responses were observed in modulated masking in terms of the behavior of CAEPs. Decreased latency and increased amplitude of cortical measurements with the use of modulated noise indicate a lower masking effect of this noise in cortical auditory processing, evidencing the masking release phenomenon.

## Introduction

Speech signal recognition is an important aspect related to the understanding of speech, and one of the conditions that can be unfavorable to this decoding process is the presence of competitive noise, which can degrade speech or make it difficult to process. 1 The presence of noise or sound, either before, during or after an initial stimulus, can generate a reduction in the perception sensitivity of such stimulus, causing a change in its sound threshold; such an event is known as “temporal masking.” 2 Although masking caused by background noise can interfere with the sensitivity of recognition of the target sound, acoustic fluctuations of these noises enable the understanding of sound/speech to occur. 3

Background noise fluctuations, both in intensity and frequency, cause favorable changes in the perception of speech/sound acoustic cues in the presence of masking, when compared with situations in which the background noise does not fluctuate, that is, it occurs steadily. 4 Some studies investigated the effect of such noise oscillations on speech recognition using behavioral methodologies and called it the masking release phenomenon 5 or benefit of modulated masking (BMM), which has been translated into Brazilian Portuguese as *benefício da modulação do mascaramento* .

Although signal-to-noise perception has been studied extensively using behavioral methodologies, 3 4 6 the neural encoding of these signals in humans still needs further investigation. Considering that electrophysiological measures, such as cortical auditory evoked potentials (CAEPs) may present different responses when acquired by stimuli presented in different types of background noise, indicating a differential effect of the type of masking on the responses, 7 the investigation of the temporal characteristics of noise, such as modulation, acquired relevance in electrophysiology.

Studies on the performance of electrophysiological tests of temporal processing have shown important clinical relevance. Such methodologies are not only complementary, but have the potential to assess temporal processing skills in participants who are unable to provide reliable behavioral responses. 8 9 Investigations on CAEPs make it possible to evaluate the entire auditory system, from the brainstem to the auditory cortex, and CAEPs can be obtained through different types of stimuli. 10 Moreover, studies on the auditory cortical wave complex (P1-N1-P2) provide important information on the neural processes responsible for speech perception, maturation of the auditory system, as well as the quality of auditory information processing. 11

Considering CAEPs an important measure to obtain objective auditory responses, the investigation of its application with modulated masking enables the assessment of analysis parameters of the masking release phenomenon in individuals with normal hearing, providing relevant findings regarding the impact of this phenomenon on temporal processing and related skills. In addition, such investigation aims to provide understanding of speech perception disorders in noise. 9

Considering the importance of studying cortical auditory measures with modulated maskers for a better understanding of the processes involved in speech recognition in noisy environments, in addition to the temporal processing skills involved in this aspect, the present review is considered relevant. The goal of the current study was to investigate the behavior of CAEPS with masking with and without modulation in the normal-hearing population, through a systematic review of the literature.

## Review of the Literature

The present systematic review was registered and published on the International Prospective Register of Systematic Reviews (PROSPERO) platform under registration number CRD42022315931, and all stages of development of the current study followed the methodological recommendations of the Preferred Reporting Items for Systematic Reviews and Meta-Analyses (PRISMA) statement. 12

### Research Strategy

The question that guided the conduction of the present research was: Is there a difference in the behavior of CAEPs with masking with or without modulation in the normal-hearing population?

The Population, Phenomenon of Interest, Context (PICo) strategy, 12 used to structure the question, was defined as follows: population (P): young people, adults, and elderly individuals, normal-hearing people; phenomenon of interest (I): performance of the CAEP with modulated masking; and context (Co): response of CAEP measures to modulated masking.

A search was performed in the Medical Subject Headings (MeSH) and Health Sciences Descriptors (Descritores em Ciências da Saúde, DeCS, in Portuguese) systems to define the descriptors to be used in the bibliographic survey; such descriptors were crossed using the Boolean operators “AND” and “OR.” After selecting the descriptors, the following search strategy was developed: ( *Young Adult* OR *Young Adults* OR *Adult* OR *Adults* OR *Middle-Aged* OR *Middle Age* OR *Aged* OR *Elderly* ) AND ( *Noise* OR *Noises* OR *Perceptual Masking* OR *Perceptual Maskings* OR *Masking Release* OR *Modulated Maskers* OR *Modulated Noise* ) AND ( *Evoked Potentials, Auditory* OR *Long-Latency Auditory Evoked Potentials* OR *LLAEP* OR *CAEP* OR *P1-N1-P2 Complex* OR *Cortical Auditory Evoked Potentials* OR *Event-Related Potentials, P300* ).

Between July and August 2022, a bibliographic survey was conducted, with a new search in May 2023 to update the review research. The search was performed in the following databases: Web of Science, Medical Literature Analysis and Retrieval System Online (MEDLINE) via PubMed, Cochrane Library, Latin American and Caribbean Health Sciences Literature (LILACS), Scientific Electronic Library Online (SciELO), and Embase, also including the following gray literature databases: OpenGrey, DissOnline, OAlster and Google Scholar. During the bibliographic survey, restrictions regarding language and date of publication of the study were not applied.

### Selection Criteria

Regarding the eligibility criteria of the studies, the review included original observational articles that performed CAEP in normal-hearing individuals, using modulated and unmodulated masking for auditory stimulation, in the young, adult, or elderly population. Case reports, letters to the editor, book chapters, animal studies, conference abstracts, and duplicate articles, as well as studies conducted with individuals presenting history of neurological and/or psychiatric diseases, cognitive deficits, and any type of hearing disorder or complaint, were excluded.

### Selection of Studies

The search and selection of studies were performed blindly by two independent reviewers with experience in hearing electrophysiology. The first stage of study selection consisted in reading the title and abstract, in digital format, of all identified articles. Those that met the inclusion criteria were selected for the next stage. In the second stage, after excluding duplicate studies, the full text of the selected articles was read, with the identification of those that met the objective of the review. Discrepancies regarding the selection of studies were discussed between the reviewers at the end of each step, aiming to reach a consensus and, in the absence of agreement, a third evaluator was consulted.

### Data Extraction

To synthesize the information from the included studies, a previously prepared Excel (Micrisoft Corp., Redmond, WA, United States) spreadsheet was used by the reviewers, who independently extracted the following data: article title, authors' names, year of publication, country, type and objective of the study, sample size, age group of the sample, auditory/cognitive assessment, CAEP performance parameters, results of latency, amplitude and electrophysiological threshold measurements of cortical components, main conclusions provided by the studies, and limitations. A third reviewer checked the extracted data to assess accuracy. All disagreements and discrepancies were resolved by discussion among the reviewers.

### Quality Assessment

The quality of the studies included in the review was analyzed using the Newcastle-Ottawa Scale (NOS) assessment instrument adapted for cross-sectional studies, 13 and two reviewers independently assessed the articles based on the following items: 1) representativeness of the sample; 2) sample size; 3) response rate; 4) determination of exposure (risk factor); 5) adequacy of research design and study analysis (control for confounding factors); 6) evaluation of results; and 7) appropriate statistical analysis. Then, scores were assigned based on three main components: selection (0 to 5 points); comparability (0 to 1 point); and results (0 to 3 points), and the maximum score was limited to 9 points/asterisks (*), representing high methodological quality. The discrepancies found in the analysis of the quality of the studies were resolved by consensus between the reviewers, and in case of persistent disagreement, the assessment was performed by a third reviewer. No studies were excluded from the present review based on the assessed risk of bias.

### Statistical Analysis of Quantitative Data

The results of each study were expressed as mean and standard deviation (SD) variables. For the continuous variables, the weighted mean difference was used to test the general effect. The randomized effect model was chosen, using the inverse variance method (this measure of variability is directly related to the sample size, that is, the larger the size, the smaller the estimated variability and, consequently, the greater the weight of the study in estimating the meta-analytical measure) and 2-tailed 95% confidence intervals (95%CIs).

Higgins statistics (I ^2^ ) was used to assess the homogeneity across studies; low heterogeneity was established if I ^2^ < 50%, and moderate and/or high heterogeneity, if I ^2^ ≥ 50%. Data were entered into an Excel spreadsheet, and statistical analyses were performed with the Revman 5.4 free software, using the meta package.

## Review Results

**Fig. 1:**
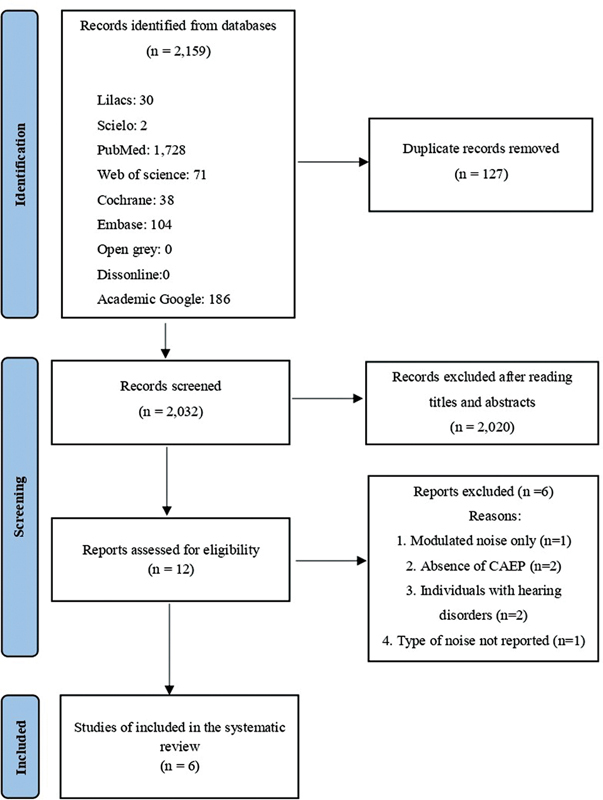
Flow diagram of the selection of articles.

A total of 2,159 studies were identified in the initial database search. After the identification stage, excluding duplicates, and screening the articles, 12 studies remained and were submitted to full-text analysis. After full-text reading, 6 studies were excluded due to the use of noise as the main and only stimulus (1), absence of cortical auditory results (2), study population with hearing disorders (2), and lack of information regarding the type of noise (1). In the end, six studies met all eligibility criteria and were included in the present review. All stages of identification and selection of studies are detailed in Fig. 1 .

In the quality assessment through the adapted NOS, the scores of the studies ranged from 5 to 7 points, and we identified that out of the six studies analyzed, five 14 15 16 17 18 had high methodological quality and one study 19 was identified as having moderate quality. All evaluated studies had low scores in the sample selection component, for they did not describe the sampling strategy neither did they jusstify the sample size. The evaluation of the “risk factor” in the selection component was the criterion with the greatest variability among studies, being scored by the description of exposure to the factor, that is, by the report of how exposure to types of noise was determined among participants. The study 19 evaluated as having moderate methodological quality was not scored on this selection criterion, which determined the final score. The evaluated studies obtained better and equal scores in the comparability and results components, in which they showed adequacy in the comparison between groups, determined by a factor (type of noise) and by the description of the evaluation of results and data analysis in a clear and appropriate way.

It is important to reinforce that regardless of the final judgment contained in the table, studies present, by nature, a high risk of bias due to non-randomization during the selection of their research subjects ( Table 1 ).

**Table 1:** Quality assessment of studies according to the adapted Newcastle-Ottawa Scale

	Selection				Comparability	Results		
Studies	Sample representativeness	Sample size	Answer rate	Risk factor	Adequacy of design and analysis	Evaluation of results	Appropriate statistical analyses	Score
Androulidakis and Jones (2006) 19	–	–	*	–	*	**	*	5
Epp et al. (2013) 14	–	–	*	**	*	**	*	7
Zhang et al. (2014) 15	–	–	*	*	*	**	*	6
Maamor and Billings (2017) 16	–	–	*	**	*	**	*	7
Tanner et al. (2019) 17	–	–	*	*	*	**	*	6
Rocha et al. (2022) 18	–	–	*	*	*	**	*	6

Note: Score classification: high quality (9–6); moderate quality (4–5); and low quality (< 4).

After the full-text reading, a detailed analysis of each article was performed, considering the baseline characteristics, the main objectives, the methodological aspects, the main results, and the conclusion. The studies included were conducted between 2006 and 2023, in Germany, 14 the United Kingdom, 19 China, 15 the United States, 16 17 and Brazil. 18 Regarding the number of study participants, a minimum of 10 and a maximum of 30 individuals of both sexes were found, except for one study, 14 which did not specify the sex of the participants. The studies were conducted with young and adult participants, aged 19 to 55 years, and only one study 16 included elderly subjects.

As for the auditory profile of the participants, five 14 15 16 17 18 out of the six studies reported on the audiological assessment, with the use of pure tone audiometric thresholds. A variation in the evaluation parameters used could be observed: two studies 15 17 included the thresholds of frequencies from 250 Hz to 8.000 Hz, while two other studies 14 16 considered frequencies up to 4,000 Hz. In addition, the threshold for the frequencies surveyed also varied, being considered normal when < 25 dB SPL, 15 16 ≤ 20 dB SPL, 17 < 20 dB SPL, 18 and ≤ 15 dB SPL. 14

Regarding the objective of the studies included, all of them aimed to investigate the effect of different maskers, including the modulated type, on the representation of CAEP; however, two studies 17 18 focused on obtaining the threshold electrophysiological analysis under different masking conditions. Table 2 shows the summary of baseline information for each study.

**Table 2:** Basic descriptions of the included studies

Author/year	City and country	Study type	Sample/gender	Age group	Clinical audiological condition	Objective
Androulidakis and Jones (2006) 19	London, United Kingdom.	Cross-sectional observational.	10 participants (5 men and 5 women).	24 to 55 (mean = 34) years.	Participants without hearing problems and with no previous history of neurological hearing conditions.	To investigate the difference between unmodulated and comomodulated maskers using event-related potentials.
Epp et al. (2013) 14	Magdeburg, Germany.	Cross-sectional observational.	10 participants.	21 to 37 years.	Participants without a history of hearing difficulties (pure tone audiometric thresholds ≤ 15 dB HL between 250 Hz and 4,000 Hz).	To investigate the correlation of the audibility of a noise masked tone in auditory evoked potentials.
Zhang et al. (2014) 15	Beijing, China.	Cross-sectional observational.	12 participants (8 men and 4 women).	19 to 25 (mean = 22.3) years.	Participants with normal hearing (audiometric thresholds < 25 dB HL between 250 and 8,000 Hz).	To examine how the initial cortical representation of speech is affected by different types of maskers.
Maamor and Billings (2017) 16	Portland, United States.	Cross-sectional observational.	30 participants (12 men and 18 women).	10 young people (mean age: 27.1 years). 10 older individuals (mean age: 67.2 years). 10 older subjects with mild to moderate sensorineural hearing loss (mean age: 68.8 years).	The 20 individuals with normal hearing had thresholds < 25 dB HL, bilaterally up to 4,000 Hz.	To determine the effects of noise type, SNR, age and hearing status on CAEP for speech sounds.
Tanner et al. (2019) 17	Chapel Hill, United States.	Cross-sectional observational.	23 participants (9 men and 14 women).	Mean = 24 years.	Participants with normal hearing (audiometric thresholds ≤ 20 dB HL in octave frequencies from 250 Hz to 8,000 Hz).	To obtain electrophysiological masking release using CAEPs with steady and modulated speech stimulus and masking.
Rocha et al. (2022) 18	Pernambuco, Brazil.	Cross-sectional observational.	14 participants (5 men and 9 women).	19 to 28 (mean = 23) years.	Participants with normal hearing (audiometric thresholds < 20 dB HL between 500 Hz and 4,000 Hz. Presence of type-A tympanometric curve and presence of ipsilateral and contralateral reflexes.	To analyze the effect of masking on the CAEPs with speech stimulus in young adults.

**Abbreviations:** CAEP, cortical auditory evoked potential; SNR, signal-to-noise ratio.

The cortical components investigated in the studies consisted of the P1-N1-P2 complex, 16 17 18 as well as a separate analysis of the amplitude and latency of the N1 and P2 components. 14 15 19

Exept for the two studies 16 18 which used monaural presentation of signals to the right ear, all of the other studies used both ears for the acquisition of cortical responses, through insert phones. In addition, half of the studies 14 17 19 mentioned the use of silver chloride (Ag/AgCl) electrodes to record potentials.

In the condition of use of modulated masking in cortical auditory responses, the use of amplitude-modulated noise with different modulation rates was observed for each study. The increasing modulation rates used were of 6.25 Hz, 17 17.5 Hz, 19 25 Hz, 17 18 between 50 Hz and 150 Hz, 15 400 Hz, 16 and 700 Hz. 14 The modulated noise intensity in each study ranged from 30 dB SPL to 80 dB SPL. Two studies 17 18 oscillated the intensity of the presentation of modulated noise between 30 dB SPL and 65 dB SPL. Table 3 shows the description of the methodological parameters used in each study.

**Table 3:** Description of methodological parameters, results, and conclusion of the included studies

Studies	Methodological parameters						Main results	Conclusion
	Recording equipment	Stimulus	Noise	Form of presentation	Acquisition parameters	Registered potentials		
Androulidakis and Jones (2006) 19	Virtual oscilloscope (Pico Technology, St Neots Cambridgeshire, United Kingdom).	Pure tone of 200 ms, 61 dB SPL, and frequency of 1 kHz.	a) Unmodulated random noise (80 dB SPL; broadband: 0–20 kHz). b) Random noise modulated in amplitude by a square wave of 17.5 Hz.	Electromagnetic earphone (Sanyo, PH230, Moriguchi, Osaka, Japan) in both ears.	Six silver electrodes in Fpz, Fz, Cz, and Pz C3 and C4 (impedance < 5 kΩ). Window (700ms); filter (1 Hz and 200 Hz).	Amplitudes and latency of the N1 and P2 components.	The N1 and P2 components were statistically higher and earlier in the modulated noise condition compared with the unmodulated noise condition. The latencies were strongly determined by whether the tones coincided with the rise or fall of the modulated masking envelope (instant noise level was low).	Masking release is correlated with waves N1 and P2. Furthermore, it is possible that the responses of these components to modulated and unmodulated masking can assess auditory temporal resolution processes, which are a crucial part of complex sound perception.
Epp et al. (2013) 14	Recording cortical potentials with EEG, with measurement (BIOSEMI ActiveTwo, Amsterdam, Netherlands) and attenuation system (Tucker-Davis Technologies, Alachua, FL, United States).	300 ms pure tone, 70 dB SPL and 700 Hz frequency.	Wideband noise (1,000ms) presented at different SNR randomly (-15, -5, +5, +15 dB) and in two conditions: a) noise modulated at 700 Hz with intensity fluctuation centered on the signal; b) non-modulated noise with frequencies not centered on the signal (300, 400, 1,000 and 1,100 Hz).	ER-2 insert earphones (Etymotic Research, Inc., Elk Grove Village, IL, United States) in both ears.	64 silver chloride electrodes, with the CZ electrode as a reference. The electrodes (A1, A2 and Iz) recorded the peaks of auditory potential responses. Window (700 to 1,250ms); filter (0 and 200 Hz).	Amplitudes of components N1 and P2.	The N1 amplitude showed very similar values for both types of masking (modulated and unmodulated). P2 amplitudes were higher for the modulated masking condition. Higher peak-to-peak amplitude of N1-P2 in modulated masking condition.	The P2 component is a candidate for an objective measure of audibility in the human auditory system. The P2 amplitude is consistent with the psychoacoustic findings of masking release.
Zhang et al. (2014) 15	Recording of cortical potentials (64-channel SynAmps, Compumedics NeuroScan, Melbourne, Australia).	a) Syllable /bi/ (target stimulus) of 474 ms, 60 dB SPL and frequency of 254 Hz. b) Syllable /di/ (frequency of 258 Hz) as a stimulus to probe the participants' attention.	a) Constant/steady noise. b) Modulated speech spectrum noise (SNR of −4 dB) and 50–150 Hz. c) Noise from two speakers (informational)	Insert headphones in both ears.	Six electrodes, being recorded at the Cz site, amplitudes and latencies of the N1/P2 components. Window (1,000–1,200ms); filter (0 Hz and 200 Hz).	Amplitudes and latency of the N1 and P2 components.	Under the conditions of passive and active listening, separately, the amplitudes were significantly higher in the conditions of steady and modulated masking, compared with the condition of informational masking. As for latencies, N1/P2 shortening was observed in the presence of constant noise in the passive listening condition and in the face of informational noise in the active listening condition.	Informational noise induced a much greater masking effect in the N1/P2 complex, suggesting that this type of masking, induced by irrelevant speech of speech signals, occurs in the early stages of cortical processing. Masking release should not be associated with attentional processes; it can be explained as a neurophysiological process.
Maamor and Billings (2017) 16	Recording of cortical potentials (Synamps RT/Scan, Compumedics NeuroScan, Melbourne, Australia) and a 64-channel electrode cap.	Syllable /ba/ and /da/, 150 ms and 65 dB SPL. Passive oddball paradigm, presentation of the pattern /ba/ of 0.8 and the deviant /da/ of 0.2.	Three types of background noise at three different SNRs: -3, 3 and 9 dB. All the noises were low-pass filtered at 4,000 Hz. a) Continuous speech noise. b) Modulated noise. c) Babbling of 4 speakers.	The signals were presented monaurally to the right ear.	The reference electrode (Cz) was located at the vertex and the ground electrode was placed on the forehead. Window (100–1,000ms); filter (0 Hz and 100 Hz).	Amplitudes and latency of components P1, N1, and P2.	A systematic decrease in amplitude and increase in latency in the cortical components was observed in the continuous noise condition, given the effect of SNR. Modulated noise contains spectral energy similar to continuous noise, but with variations in the temporal envelope.	The little interference of the SNR effect in the modulated noise condition can be explained by the fluctuations/gaps in the modulated noise, making the signal audible, regardless of the noise level. It is concluded that the spectrotemporal characteristics of signals and noises play an important role in determining the morphology of neural responses.
Tanner et al. (2019) 17	Recording of cortical potentials (SynAmp RT, Compumedics NeuroScan, Melbourne, Australia) synchronized with the Tucker-Davis Technologies (Alachua, FL, United States) stimulation system.	Syllable /ba/ of 80 ms, 40 dB SPL and frequency of 1 kHz.	Speech spectrum noise in 4 conditions: a) low steady noise (30 dB SPL); b) high steady noise (65 dB SPL);. c) modulated noise at an intensity of 65 dB SPL and 30 dB SPL with a modulation rate of 6.25 Hz (slow modulated); d) modulated noise at an intensity of 65 dB SPL and 30 dB SPL with a modulation rate of 25 Hz (fast modulated).	Electromagnetically shielded earphones (ER2, EtymoticResearch, Inc., Elk Grove Village, IL, United States ), in both ears.	4 silver chloride electrodes: A1 and A2 (ear lobes), Fpz (high forehead line) ,and Cz (skull vertex). (Impedance ≤ 5 kΩ and ≤ 3 kΩ between electrodes.) Window (-100 to +300ms); filter (1 Hz and 100 Hz).	Amplitude and latency of the P1-N1-P2 complex.	The mean electrophysiological threshold in modulated masking was ∼ 13.5 dB lower than in the steady masking, resulting in masking release. It was observed that, for the different types of noise, as the SNR decreases, the general amplitude of the P1-N1-P2 response decreased, and the peak latencies increase – mainly that of N1.	Electrophysiological measures of masking release using CAEP with speech stimuli correspond to behavioral estimates for the same stimuli. It is suggested that objective measures based on electrophysiological techniques can be used to reliably assess aspects of temporal processing capacity.
Rocha et al. (2022) 18	Recording of cortical potentials (IHS equipment, Intelligent Hearing Systems, Miami, FL, United States) with the Tucker-Davis Technologies (Alachua, FL, United States) stimulation system.	Syllable /ba/ of 80 ms, 65 dB SPL and presentation rate of 3.8.	Speech spectrum noise in 3 conditions: a) weak steady noise (30 dB SPL); b) strong steady noise (65 dB SPL); c) modulated noise at an intensity of 65 dB SPL and 30 dB SPL with a modulation rate of 25 Hz.	Electromagnetically-shielded earphones (ER2, EtymoticResearch, Inc., Elk Grove Village, IL, United States) in the right ear.	4 electrodes: A1 and A2 (ear lobes), Fpz (high forehead line) and Cz (skull vertex). (Impedance ≤ 5kΩ.) Window (512ms); filter (1 Hz and 30 Hz).	Amplitude, latency, and morfology of the P1-N1-P2 complex.	P1 and N1 showed higher latencies in the stable noise condition. More robust amplitudes of the P1-N1-P2 complex were recorded in the modulated noise condition, with statistical significance. The mean electrophysiological thresholds were higher in the stable noise condition (60 dB SPL), with an average difference of 11.7 dB higher than in the modulated noise condition.	There was a lower masking effect of modulated noise when compared with the strong steady noise condition, in the amplitude measurements of the P1-N1-P2 complex. The mean difference of 11.7 dB between the electrophysiological thresholds (under conditions of stable noise and modulated noise) was interpreted as the masking release measure.

**Abbreviations** : CAEP, Cortical auditory evoked potential; EEG, electroencephalography; SNR, signal-to-noise ratio.

Based on the results presented by the studies included in the review, we observed that three articles 16 18 19 described quantitative results regarding latency and amplitude measurements of the cortical components analyzed, and the other studies 14 15 17 reported the results of cortical responses graphically and statistically.

Table 4 describes a quantitative synthesis of the data extracted from the studies. Considering the heterogeneity of the analysis conditions of the cortical components used in the studies, this quantitative synthesis of the data was necessary for the development of the meta-analysis.

**Table 4:** Summary of quantitative results of cortical auditory potentials of the included studies

Studies		CAEP latency (ms)			CAEP amplitude (µV)		
	Type of masking	P1	N1	P2	P1	N1	P2
Androulidakis and Jones (2006) 19	Steady	–	165.2 (11.3)	263.6 (16.4)	–	1.9 (0.5)	3.2 (0.7)
	Modulated	–	126.3 (10.3)	204.5 (17.7)	–	2.9 (0.6)	4.9 (1.1)
Zhang et al. (2014) 15	Steady	–	168.8	237	–	2.49	2.62
	Modulated	–	157 -	240.7 -	–	2.19 -	2.19 -
Maamor and Billings (2017) 16	Steady	86 (10.3)	151 (10.4)	227 (26.9)	0.45 (0.2)	1.84 (0.4)	0.87 (0.5)
	Modulated	74 (10.3)	146 (12.8)	243 (35.5)	0.55 (0.3)	1.39 (0.3)	0.65 (0.3)
Rocha et al. (2022) 18	Steady	81.2 (33.5)	145.2 (34.7)	196.2 (35.1)	4.0 (1.1)	1.7 (1.2)	1.8 (1.1)
	Modulated	73.0 (16.8)	140.6 (18.5)	211.2 (16.8)	5.6 (1.1)	4.1 (1.8)	4.7 (2.1)

**Abbreviation:** CAEP, cortical auditory evoked potential.

**Note:** Results expressed as mean and standard deviation values.

**Fig. 2:**
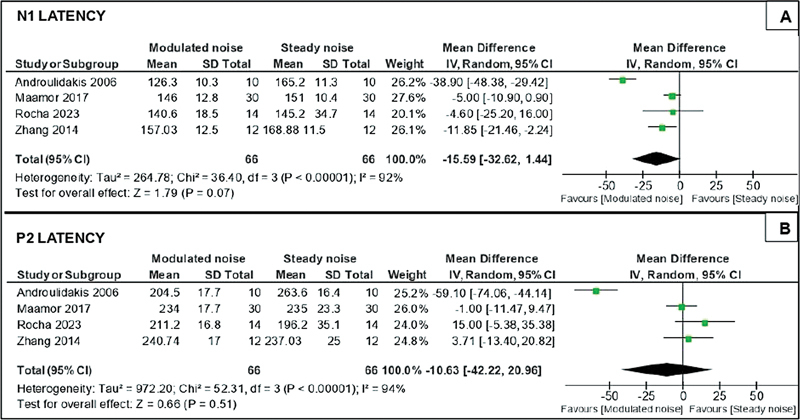
Quantitative analysis of N1 and P2 latency measurements.

**Fig. 3:**
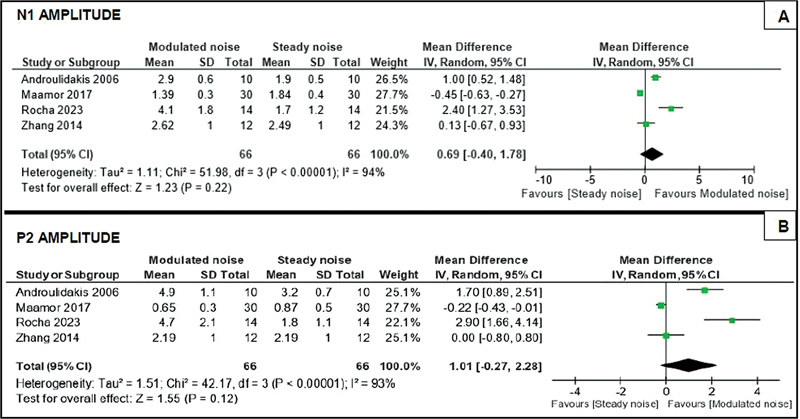
Quantitative analysis of N1 and P2 amplitude measurements.

The results obtained with the meta-analysis of quantitative data are shown in Figs. 2 and 3 , which express the quantitative analysis of latency measures and amplitude of cortical components N1 and P2. It was not possible to analyze the P1 component due to insufficient quantitative data in the selected studies. In total, 4 studies 15 16 18 19 were included in the meta-analysis, comprising 66 participants who underwent the CAEP assessment with and without modulated masking.

Based on the meta-analysis of the data, high heterogeneity (I ^2^ ≥ 92%) was observed in the included studies 15 16 18 19 for the cortical components (N1 and P2) and measures (latency and amplitude). In the analysis of the latency ( Fig. 2 ) and amplitude ( Fig. 3 ) measures of the N1 and P2 components, the forest plot graphs indicated that the effect of the meta-analysis was not statistically significant ( *p* > 0.05) among the masking groups analyzed for both the cortical components, interpreted by the diamond figure that touches and crosses the vertical line of nullity in all graphs of the meta-analysis. Therefore, it was not possible to determine the favorability among the masking conditions studied for the latency and amplitude measurements of the cortical components analyzed. The low number of studies that made up the meta-analysis made the use of heterogeneity exploratory techniques unfeasible due to the limitations in determining significant estimates of the results.

## Discussion

The evaluation of cortical auditory measures with the use of modulated maskers is necessary for a better understanding of the processes involved in speech recognition in noise situations, as well as of the temporal processing skills involved in this aspect. Therefore, the current review aimed to investigate the behavior of CAEPs with masking with and without modulation, in the normal-hearing population, through a systematic review of the literature.

The study of the effect of noise on electrophysiological hearing responses enables us to understand the processes involved in speech recognition in situations of competitive noise and the performance of individuals' temporal processing skills. Considering that the central auditory system (CAS), specifically the cortex, plays an important role in signal-to-noise coding, 20 and that this condition is also affected by factors related to the spectrotemporal properties of these signals, 7 the analysis of the behavior of the CAS in different conditions of competitive noise, through the CAEP measures, results in indispensable information on the variations of performance among normal-hearing individuals.

Modulated noise, in turn, used competitively by behavioral auditory measures, has been shown to generate better results in terms of the perception of speech signals and sounds 21 22 23 24 when compared with steady noise, or without modulation. However, in the electrophysiological domain, the investigation of the effects of this noise is still limited, which justifies the number of studies included in the present review.

The current review showed that to observe the CAEP behavior in modulated noise, the studies used comparisons with other conditions of unmodulated noise, such as steady broadband noise, 14 19 steady speech spectrum noise, 15 16 17 18 and informational noise, also called babbling of two 15 and four speakers. 16 In addition to comparing the different types of noise in cortical auditory responses, two of the studies 14 16 used different types of signal-to-noise ratio (SNR) in a random way, to observe the effect of this change in cortical responses.

Based on the methodological parameters used to acquire the CAEP, four of the studies used speech stimuli, which were the syllables /ba/ 16 17 18 and /bi/, 15 as the main stimulus, and the syllables /di/ 15 and /da/ as a deviant stimulus. 16 The other studies used pure tones to evoke cortical responses, with a fixed intensity of 61 dB SPL 19 and 70 dB SPL. 14 Variation in stimulus intensity was also observed among studies that used speech signals, with intensities of 40 dB SPL, 17 60 dB SPL 15 and 65 dB SPL. 16 18 The speech stimulus duration among the studies ranged from 80 ms to 474 ms; pure tone stimuli ranged from 200 ms to 300 ms.

It seems relevant to emphasize that in the CAEP recording and analysis, different types of stimuli can be used, such as tonal stimulus and speech stimulus; however, the literature shows that the use of speech stimuli makes it possible to analyze the processes underlying neural encoding and decoding for complex signals, 25 26 27 which may help in the assessment of speech auditory processing.

Regarding the use of filtering of captured cortical electrical responses, no standardization was observed for this acquisition parameter; however, three of the studies included used filters up to 200 Hz, 14 15 19 2 articles 16 17 used the filter of up to 100 Hz, and only 1 study used a 30-Hz filter. 18

The different parameters used for CAEP acquisition reported in the current review show some challenges that are still present in the choice of protocols to capture cortical auditory responses and their relationship with normality markers that can be applied in a standardized way in the clinical context.

As for the CAEP record, the study by Androulidakis and Jones 19 (2006) described that the N1 and P2 components were statistically larger (in terms of their morphology) and earlier in the modulated noise condition compared with the non-modulated/steady noise condition. In addition, an increase in these responses for the P1-N1-P2 complex was pointed in the latency results in the condition of continuous/steady noise when compared with situations of modulated and informational noise. 16 Rocha et al. 18 (2022) also identified an increase in the latency values for the P1 and N1 components in the steady masking condition when compared with modulated masking.

These results support the idea that cortical auditory potentials indicate a differential effect of the type of masking on their responses when recorded in different competitive noises. 7 In view of this, considering that background noise commonly reduces the amplitude responses and increases the latency evoked by a sound, 28 we concluded that the type of masking has a significant influence on these responses.

Moreover, regarding the behavior of the latencies of the cortical components, their decrease observed in modulated masking was determined by the fact that the acoustic signals coincide with the rise or fall of the modulated noise, in which the instantaneous noise level is considered low, generating a shorter time in the cortical auditory response. 19 This concept corroborates the findings in the literature that claim that despite the masking caused by background noise, fluctuations in modulated noise generate a better perception of acoustic speech cues in individuals with normal hearing due to a more favorable “speech-to-noise” ratio. 3 4

With regard to the amplitude of cortical responses, the studies 14 16 17 18 19 showed that greater amplitudes were obtained with the use of modulated masking noise, considering a better magnitude of cortical activity in the processing of verbal stimuli in this type of noise, when compared with steady noise. This result can be explained based on findings that show that modulations in noise intensity cause a decrease in the signal-to-noise ratio and consequently generate an increase in the amplitude of the evoked response 24 .

Some studies have also reported that responses from cortical components, specifically N1 and P2, may be consistent with psychoacoustic findings regarding the effect of noise modulation on auditory discrimination, evidencing masking release, 14 19 and that this phenomenon (masking release) should not be associated with attentional processes. 15

Regarding the electrophysiological threshold investigated in different masking situations (steady and modulated), 2 studies reported that this threshold was lower in modulated noise, with a difference of approximately 13.5 dB 17 and 11.7 dB 18 lower when compared with steady noise; this value was considered the quantitative expression of the masking release. 17

The correspondence between the CAEP electrophysiological measures and the behavioral estimates pointed out in the studies resulted in the authors' 17 18 suggestions that objective tests based on electrophysiological methods could be used to reliably assess aspects of temporal processing capacity.

Regarding aspects related to the use of modulated noise as masking in the acquisition of more audible signal, regardless of the noise level; and that when compared with steady noise, both have similar spectral energy. However, the temporal envelope variations in the modulation determine the difference in the morphology of neural responses. 16

As for the evaluation of the methodological quality of the articles, although five studies 14 15 16 17 18 were considered of high quality, it is important to highlight that all of them presented selection bias, not mentioning information about the sample of research subjects, confident for the increased risk of bias.

The cross-sectional observational design of the studies included in the present review has a body of evidence with a high rate of bias, which emphasizes the need for more research on the topic of this review, with more careful methodologies to improve the quality of the studies. However, it is worth mentioning that many important data would be discarded if the selection criteria for studies, in systematic reviews, were necessarily studies with high level of evidence, since in the field of audiology, there are few more robust studies, with greater control of bias, such as controlled and randomized clinical trials.

In the meta-analysis, it was not possible to measure the effect of the performance of the masking conditions analyzed, since the results demonstrated high heterogeneity among the included studies. This high rate of disparity found in the meta-analysis can be explained by the variability in the methodological parameters obtained in each research, in addition to the low number of studies included in the review, being important points of high influence in the meta-analysis. It is important to highlight that the number of studies included in the present review, added to their lack of methodological information and quantitative results, can be pointed out as a limitation of the current systematic review, reinforcing the importance of carrying out higher-quality studies regarding the effect of modulation of noise in cortical auditory potentials.

## Conclusion

The results of the present review show that the type of masking has an influence on cortical auditory behavior, indicating a different effect on neural posture regarding CAEP responses. Modulated noise as masking in the CAEP record generated statistically higher and earlier responses compared with non-modulated/steady noise, evidenced by the results obtained in the meta-analysis with subgroup analysis. The behavior of CAEP with better responses in modulated masking, indicating a smaller effect of the masking in cortical auditory processing, evidences the phenomenon of masking release. Due to the variation in the methodological parameters used to evoke the CAEP observed the included studies, it is not possible to generalize the findings of the present review.
